# Real-world use of once-weekly semaglutide in patients with type 2 diabetes at an outpatient clinic in Spain

**DOI:** 10.3389/fendo.2022.995646

**Published:** 2022-09-16

**Authors:** Maria Dolores Garcia de Lucas, Jose Pablo Miramontes-González, Beatriz Avilés-Bueno, Ana Isabel Jiménez-Millán, Francisco Rivas-Ruiz, Luis M. Pérez-Belmonte

**Affiliations:** ^1^ Department of Internal Medicine, Hospital Costa del Sol, Marbella, Spain; ^2^ Department of Internal Medicine, University Hospital Rio Hortega, Valladolid, Spain; ^3^ Nephrology Department, Hospital Costa del Sol, Marbella, Spain; ^4^ Endocrinology Department, Hospital P, Puerto Real Hospital, Cadiz, Spain; ^5^ Research Unit, Hospital Costa del Sol, Marbella, Spain; ^6^ Internal Medicine Department, Regional University Hospital of Málaga, Biomedical Research Institute of Málaga (IBIMA), University of Málaga (UMA), Málaga, Spain

**Keywords:** once-weekly semaglutide, T2DM, HbA1c, body weight, real-world use

## Abstract

**Objectives:**

To investigate the use of once-weekly semaglutide in a real population of people with type 2 diabetes mellitus (T2DM) in three Spanish hospitals.

**Method:**

An observational, retrospective and multicenter clinical study was designed that included 166 participants with T2DM, distinguishing between a group naïve to GLP-1RA (n=72) and another switching from another GLP-1RA (n=94), all managed in the outpatient clinical setting. The primary endpoint was the change in HbA1c from baseline to the end of the study. The secondary endpoints included changes in body weight and the proportion of people with T2DM, achieving HbA1c <7.0% and body weight loss >5%.

**Results:**

After 24 months of follow-up, the reductions in HbA1c were -0.91 ± 0.7% (p<0.001) in the total cohort, -1.13 ± 1.38% (p<0.019) for GLP-1RA-naïve participants, and -0.74 ± 0.9% (p<0.023) for GLP-1RA-experienced participants. Body weight reductions were -12.42 ± 9.1% in GLP-1RA-naïve participants vs. -7.65 ± 9.7% in GLP-1RA-experienced participants (p<0.001). In the total cohort, 77.1% reached the objective of an HbA1c level <7%, and 12.7% reached between 7.1% and 7.5%. Additionally, 66.9% achieved a weight reduction ≥5%. Of all cohort, 90% received 1 mg of semaglutide once a week. The reported adverse events were consistent with the known safety profile of semaglutide.

**Conclusions:**

In routine clinical practice in Spain, the use of semaglutide once a week was associated with statistically significant and clinically relevant improvements in HbA1c and body weight in a wide range of adults with T2DM, without notable adverse effects, which supports real-world use.

## Highlights

- OW semaglutide in real life, as early treatment, improves glycemic control and weight loss in T2DM.
- OW semaglutide favors early comprehensive management in T2DM in real life.
- OW semaglutide is an effective treatment in GLP-1RA-naïve and GLP-1RA-experienced people.

## Introduction

The goal of treating diabetes mellitus is to prevent or delay the onset of complications, in addition to ensuring a good quality of life through adequate metabolic control and reducing the cardiovascular (CV) risk factors that accompany diabetes (1). In Spain, an estimated 13% of people have diabetes mellitus, mostly type 2 (T2DM) (2). In more than 50% of cases, other CV risk factors such as overweight or obesity, hypertension, and dyslipidemia are associated with T2DM. To achieve these goals, a wide therapeutic arsenal is available for the treatment of T2DM. However, only GLP-1 receptor agonists (GLP-1 RA) have demonstrated their multifactorial efficacy in reducing HbA1c, favoring weight loss, and improving CV survival, as demonstrated by eight randomized clinical trials of GLP-1RA in both patients with established vascular events and those at high cardiovascular risk without vascular event (3).

Semaglutide is a GLP-1 RA administered subcutaneously (once-weekly subcutaneous; OWS) and recently orally. Randomized clinical trials have demonstrated its superiority and efficacy in the sustained decrease of HbA1c and weight compared to placebo and other active comparators (canagliflozin 300 mg, dulaglutide 0.75 and 1.5 mg, exenatide extended-release 2.0 mg, insulin glargine, liraglutide 1.2 mg, and sitagliptin 100 mg) (4), as well as in association with insulin, metformin (5), or SGLT2-i (6). A low risk of hypoglycemia exists, and evidence indicates a potential kidney-protective effect (7). These valuable data from clinical trials represent a highly selected population, which often is different from that treated in clinical practice, necessitating validating these results in real-life studies. To this end, the SURE program was carried out, consisting of nine observational studies in ten countries. Real-world results on the use of OWS in clinical practice have thus been compiled, with a follow-up limited to 30 weeks, and published for five countries: Canada (8), Denmark and Sweden (9), the UK (10), and Switzerland (11).

The objective of our work was to carry out a real-life study in TD2M, either naïve or with previous treatment with other GLP-1 RAs, with OWS at 2 years, carried out at three Spanish hospitals. The study used a multidisciplinary approach, with patients treated by internists, endocrinologists, and nephrologists.

## Participants and methods

### Study design

This multicenter, retrospective, observational clinical study included 166 participants with T2DM initiating semaglutide OWS between May 2019 and December 2021. Of these, 72 participants (43.4%) were GLP-1RA-naïve (no GLP-1RA use in the previous 12 months), and 94 (56.6%) were GLP-1RA-experienced (current non-semaglutide GLP-1RA users). They were managed at the outpatient clinics of the departments of internal medicine, nephrology, and endocrinology of three Spanish hospitals in Andalusia: the Costa del Sol Hospital in Marbella, Regional University Hospital of Málaga, and the Puerto Real Hospital in Cádiz.

All cohort signed an informed consent, and the study was conducted in accordance with the Declaration of Helsinki (12) and the Guidelines for Good Pharmacoepidemiology Practices (13). The study materials were approved by the local Data Protection Agency. We collected data at baseline and after 6, 12, 18, and 24 months ± 4 weeks from the beginning of treatment in clinical practice with OWS. The physician determined the semaglutide initiation dose, dose escalation schedule, intended maintenance dose, and any changes thereafter. Diet and physical activity counseling and additional antihyperglycemic treatments were permitted as part of routine clinical practice.

### Study population

The inclusion criteria were age ≥18 years at informed consent, T2DM diagnosis ≥12 weeks before inclusion, ≥1 documented HbA1c value within 12 weeks before informed consent and treatment initiation, and glomerular filtration rate (GFR) estimated using the Chronic Kidney Disease Epidemiology Collaboration (CKD-EPI) formula of >15 mL/min/1.73 m^2^. Participants under 18 years, pregnant, with CKD stage 5, or taking part in clinical trials were excluded from the study. For GLP-1RA-naïve participants, OWS was initiated at a weekly dose of 0.25 mg, which was increased to 0.5 mg after 4 weeks. In GLP-1RA-experienced participants, OWS was initiated at a weekly dose of 0.5 mg. In both groups, the dose was further increased to 1 mg weekly after 4–8 weeks if the glycemic target was not reached and side effects did not hinder it. In addition, hypertension and dyslipidemia treatments were intensified during the follow-up period when necessary.

### Endpoints

The primary endpoint was the change from baseline to the end of study (EOS) in HbA1c (%-point). Secondary endpoints included the change from baseline to EOS in body weight (kg), proportion of participants achieving HbA1c level <7.0% at EOS, and proportion of participants achieving a weight reduction of 5% at EOS. Other secondary outcome variables included any changes in fasting blood glucose levels, body mass index (BMI), blood pressure, eGFR, UACR, lipids, and insulin dose at EOS. Hypoglycemic episodes were defined according to the American Diabetes Association criteria (1).

### Safety

Only serious adverse drug reactions and fatal events were systematically collected during the study. Cardiovascular events, hospital admissions, and other possible adverse drug effects were also collected.

### Statistical analysis

A descriptive analysis was performed using measurements of central tendency, dispersion, and position for quantitative variables and distribution of frequency for qualitative variables.

To evaluate differences between dichotomous variables concerning quantitative variables, Student’s t-test was used with versions for independent or paired samples depending on the nature of the variable. The chi-square test (or Fisher’s exact test in the case of expected frequencies less than 5) was used for qualitative variables.

A paired-sample t-test was used for quantitative variables, and McNemar’s test was used for qualitative variables, to study differences between pairs. Statistical significance was established as p<0.05. All statistical analyses were performed using SPSS v. 28.0 software.

## Results

### Baseline characteristics

**Figure 1:**
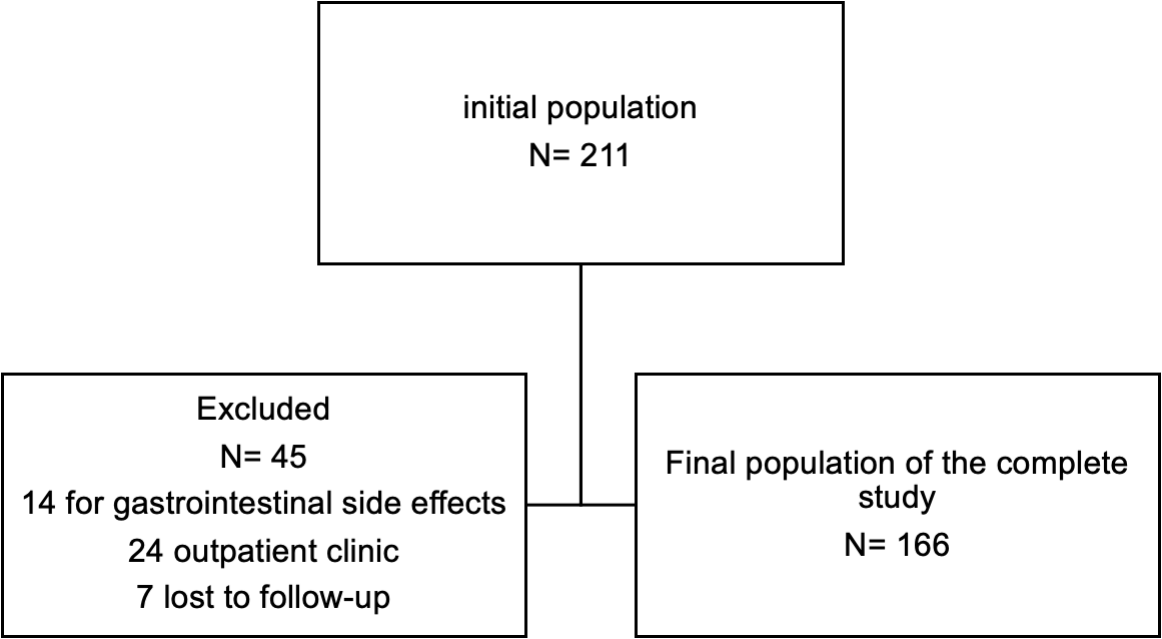
Flow chart of the study population. A total of 211 people with T2DM initiated treatment with semaglutide, of whom 166 (78.8%) completed the study on treatment with semaglutide. 45 patients were not suitable for follow-up.

A total of 211 participants with T2DM initiated treatment with semaglutide (see **Figure 1**), of whom 166 (78.8%) completed the two year-study on treatment with semaglutide. Forty-five (22.2%) were not eligible for follow-up (14 dropped out because of gastrointestinal side effects, 24 were referred from the outpatient clinic to general practice, and seven were lost to follow-up).

The clinical and epidemiological characteristics of the 166 adults included in our study are shown in **Table 1**. The mean age was 65.56 ± 11 years, 62% were males, all were Caucasian and the mean duration of T2DM was 9.95 ± 9 years. The mean body weight was 98.48 ± 16.6 kg, and the mean BMI was 35.9 ± 5.74 kg/m^2^.

**Table 1:** Baseline characteristics of patients overall.

	Total cohort	GLP−1RAnaive	GLP−1RA-experienced	P
Numbers	166	72	94	ns
Men	92 (62%)	43 (59.7%)	54 (57.4%)	ns
Age (years)	65.56±11	65.56±11	63.61±11.1	ns
Diabetes duration (years)	9.95±9	8.85±9.2	9.21±10.3	ns
Weight (kg)	98.48±16.6	97.86 ±18	97.13 ±16.3	ns
BMI (kg/m^2^)	35.88±5.74	35.81±5.7	35.92±5.8	ns
HbA1c (%)	7.50±1.40	7.93±1.40	7.50±1.40	ns
BP (systolic/diastolic) (mm Hg)	127.8±10.3/77.1±8.3	127.2±11.4/74.5±8.3	127.2±11.4/74.5±8.3	ns
Macrovascular complications	58%	47%	47%	P 0.06
Microvascular complications	68%	63%	65%	ns
≥3 ADD^b^	1.8	1.5	1.1	P 0.06
Metformin treated	51%	42%	44%	ns
Basal Insulin (IU)	43.9±22	38±23.5	44±21	ns
Prandial Insulin (IU)	30±18.7	29±24	28±21	ns

Values are mean (SD) unless otherwise specified. BMI, body mass index; GLP-1RA, glucagon-like peptide 1 (GLP-1) receptor agonis; HbA1c, glycated haemoglobin A1c. Table presents baseline characteristics for total cohort and subgroups: GLP-1RA naive and GLP-1RAexperienced. ^b^Antidiabetic drugs: metformin, dipeptidyl peptidase 4-inhibitor, sulphonylurea, SGLT2i, insulin.

Most participants (64%) had one or more complications of diabetes, the majority with both macrovascular (58%: ischemic heart disease, stroke, peripheral angiopathy) and microvascular (68%: nephropathy, retinopathy, neuropathy) complications. Of all adults, 14% were smokers, and 42.5% were ex-smokers. In both subgroups, the mean age and initial BMI were similar. However, GLP-1RA-experienced participants had more years of evolution of T2DM, more insulin use, and more complications, without the difference being significant.

### Semaglutide treatment and dose

After 24 months of semaglutide OWS treatment, 149 participants (90%) received the maximum dose of semaglutide (1 mg once weekly); of these, 64 were GLP-1RA-naïve (88% of total GLP-1RA naïve) and 85 were GLP-1RA-experienced (90.5% of total GLP-1RA-experienced). For 10%, the dose was maintained at 0.5 mg/week.

At baseline, 51% of participants received metformin, 18% dipeptidyl peptidase-4 (DPP-4) inhibitors, 56% SGLT2-i, 54.8% basal insulin, and 9% rapid-acting insulin. Of all patients, 94 (56.6%) switched from another GLP-1RA (liraglutide, dulaglutide, exenatide LAR) to OWS in the framework of this study. After 12 months, 49% were on metformin, 0% DPP-4 inhibitor, 67% SGLT2-i, 100% GLP-1RA, and 54% insulin; 81.3% took antihypertensives. The majority (95%) were on inhibitors of the renin angiotensin system (RAAS); 87.3% were taking high-potency statins, 28% ezetimibe, and 3% PCSK9; 67% were under antiaggregants or anticoagulants.

The mean number of antidiabetic drugs was decreased from 1.5 to 1.14 among the GLP-1RA-naïve participants and from 1.10 to 1.04 among the GLP-1RA-experienced participants, with statistical significance for the GLP-1RA-naïve group (p<0.001).

### Glycated hemoglobin

**Figure 2:**
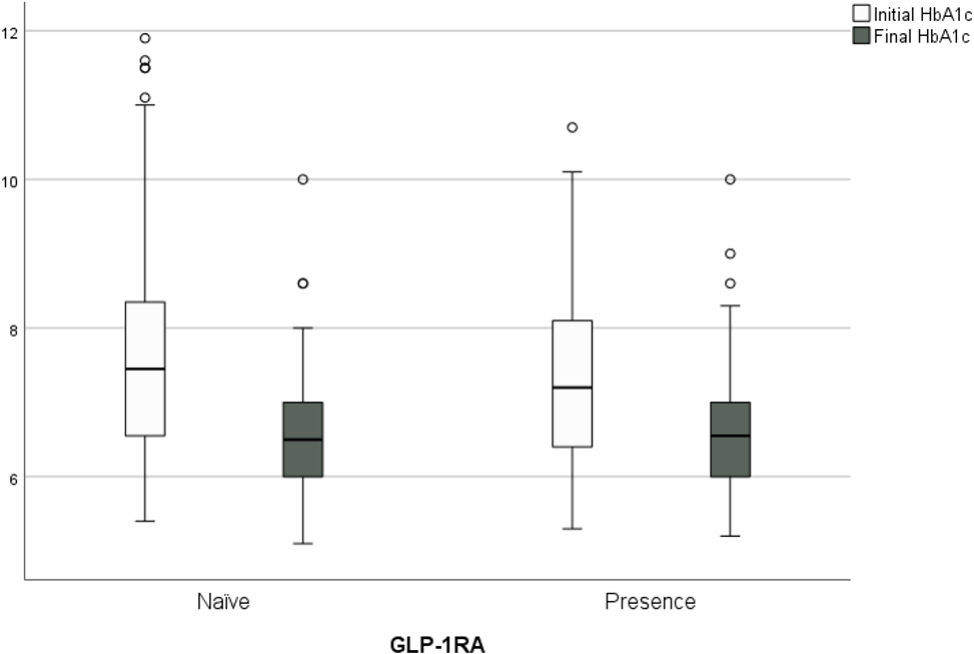
Change in HbA1c from baseline to the end of study, 77.8% of GLP- 1 RA- naïve people achieved an HbA1c of <7% at 24 months, as did 76.6% of GLP- 1RA- experienced people, with no significant difference.

Treatment with semaglutide once weekly was associated with significant decreases in HbA1c after 24 months compared with baseline in the total cohort. The mean change in HbA1c was -0.91 ± 0.7% (from 7.50 ± 1.40 to 6.59 ± 0.86, p<0.001) with 77.1% of participants achieving the target of an HbA1c level <7%, another 12.7% reaching 7.1%–7.5%, and 10.2% reaching 7.6%–8.5%. As shown in **Figure 2**, looking at the two subgroups, 77.8% of GLP-1RA-naïve participants achieved an HbA1c of <7% at 24 months, as did 76.6% of GLP-1RA-experienced participants with no significant difference. Adjustment for age, sex, diabetes duration, and number of drugs at baseline did not change the results.

### Body weight

**Figure 3:**
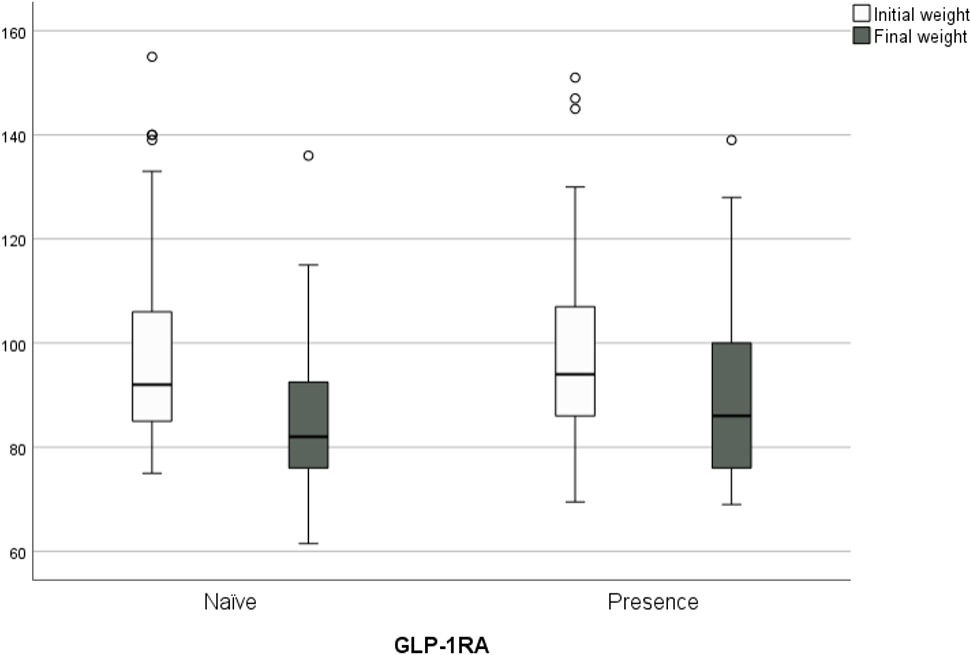
Change in body weight from baseline to the end of study (-9.72 ± 9.7 kg) (from 98.48 ± 16.6 kg to 88.76 ± 16.42 kg; p<0.001). Weight loss was greater for GLP- 1RA- naïve people (-12.42 ± 9.1 kg vs. -7.65 ± 9.7 kg; p<0.001).

Participants had a mean weight loss of -9.72 ± 9.7 kg (from 98.48 ± 16.6 kg to 88.76 ± 16.42 kg; p<0.001), with 66.9% achieving the target of -5% of total body weight and statistical significance in participants with <5 years’ evolution of T2DM (p<0.039). The loss was greater in those older than 65 years (74.4%; p<0.07), with no significant differences by sex. Adjusted for age, sex, diabetes duration, and number of drugs at baseline, weight loss was greater for GLP-1RA-naïve participants (-12.42 ± 9.1 kg vs. -7.65 ± 9.7 kg; p<0.001) as shown in **Figure 3** and men (-14.04 ± 10.35 kg; p<0.002), and more participants achieved a weight loss of >5% (76.4%; p<0.034).

At follow-up, BMI was significantly reduced (-3.68 ± 3.6 kg/m^2^; p<0.001). By subgroups, the decrease in BMI was greater in GLP-1RA-naïve participants (-4.66± 3.7 kg/m^2^ vs. -2.93± 3.5 kg/m^2^; p<0.002). we analyze the combined variable of HbA1c <7% and weight loss >5%, the goal was reached in 63.9% of the GLP-1RA-naïve participants and 51.1% of the GLP-1RA-experienced participants without significant difference, but with a clear trend in favor of naïve people.

### Insulin

In participants using insulin (n=91), the mean total basal insulin dose significantly decreased after 24 months: 43.9 UI initial vs. 29.5 UI final (-14.3 UI; p<0.001) and prandial insulin 30 UI initial vs. 14.7 UI final (-15.3 UI; p<0.002). During the 24 months of follow-up, none stopped insulin therapy. By subgroup, significantly fewer GLP-1RA-naïve participants maintained basal insulin therapy (29, 40.3% vs. 62, 66%; p<0.002), with both groups decreasing the number of units at 24 months, but the difference did not reach statistical significance.

### Other clinical outcomes

Compared with baseline, treatment with semaglutide once weekly did not result in any significant changes to either systolic blood pressure (SDP; -1.0 [-2.5; 1.1] mmHg) or diastolic blood pressure (DBP; −0.9 [−1.5; 0.5] mmHg) after 24 months in the total cohort or any of the subgroups.

At follow-up, a significant reduction was found in glucose (-29 ± 48 mg/dL; p<0.001) triglycerides (-41.8 ± 95.4 mg/dL; p<0.001), LDL cholesterol (−19.2 ± 33.3 mg/dL; p<0.001), ALT (-5.5 ± 11.7 U/L; p<0.001), and albuminuria (-47.1 ± 263.4 mg/g; p<0.011), with no significant difference in eGFR (−0.86 ± 11.4 mL/min/1.73m2; P=0.10).

### Hypoglycemia and side effects

Of the 91 participants who maintained insulin use, no change existed in the proportion reporting at least one incidence of weekly hypoglycemia between baseline (8.4%) and follow-up (8.1%; p=0.80) or in the proportion reporting at least one incidence of yearly severe hypoglycemia (baseline 0.2%, follow-up 0.2%; p=0.75). No participant without insulin therapy presented hypoglycemia.

After 24 months, eight participants (5%; three GLP-1RA-experienced and five GLP-1RA-naïve) reported gastrointestinal side effects. One participant was diagnosed with stroke and one with acute myocardial infarction.

## Discussion

In recent years, the incorporation of new treatments for T2DM has provided benefits beyond glycemic control. Of the last two therapeutic groups, SGLT2-i and GLP1-RA, the latter has been shown superior in controlling overall vascular risk. Not only linked to glycemic control or weight improvement, its benefit goes beyond improving the atherosclerotic profile of patients. Numerous clinical trials demonstrate these beneficial effects, including those published in the SUSTAIN 1–7 program with an extension between 2 and 5 years (4). At the level of real clinical practice, observational studies support these observations, such as the SURE program of nine studies in ten European countries with an average extension of 30 weeks. Independent real-life studies complement these observational studies, such as the real-life study at 52 weeks published by Hansen et al. (14). We present a real-life study at 104 weeks (2 years) carried out in three Spanish hospitals of the public network, with patients treated in outpatient clinics from primary care and other specialties.

Our results show that participants who persisted on semaglutide therapy throughout the 24-month follow-up period had a mean HbA1c reduction of 0.91%, with 77.1% of participants achieving the target of an HbA1c level of <7%, and a mean weight reduction of 9.7 kg, with 66.9% of participants achieving the target of -5% of total weight, comparable with what has been observed in the SUSTAIN and SURE studies. The achievement of these good results in just 2 years is notable, along with the fact that the population was of upper middle age and with complications from T2DM, few dropouts from treatment, and very few side effects. All this reaffirms the need in clinical practice for its early use, a fact that can also have long-term repercussions in reducing the appearance of complications and vascular events. This reiterates that early comprehensive management of T2DM will reduce its high CV mortality, as reflected in the CAPTURE real-life study (15).

The metabolic control results can be analyzed by comparing the data with the pivotal studies. If we compare the decrease in HbA1c in our study, which was -0.9%, with SUSTAIN, it was slightly lower: -1.4% with the 1-mg semaglutide dose after 49 weeks of follow-up (4). Several reasons can justify these differences. The p<7% before entering the study, unlike in SUSTAIN, whose baseline HbA1c was >8%^4^; and participants who were switched from another GLP-1AR had less HbA1c reduction than in other studies (-0.74 vs. -1.13) (16). In addition, the patients in the SUSTAIN trials, except for those in SUSTAIN 6, did not have complications from T2DM, were younger, and had a shorter duration of T2DM (7). In our cohort of 166 participants, 68% had microvascular complications, and 58% had macrovascular complications. SUSTAIN participants had a personalized follow-up program encouraging the objectives of the study and favoring adherence to therapy, tools that are not available in real-life studies. All the restrictions associated with the COVID pandemic since 2021 may have negatively affected adherence to appropriate lifestyle and therapy. Even so, the overall percentage of participants who obtained an HbA1c of <7% was slightly higher than in SUSTAIN 1 (17), 3 (18), 4 (19), and 7 (20): 77.1% vs. 74%, 67%, 73%, and 68%, respectively.

Weight loss, a key benefit of therapy with GLP-1AR, was -9.72 ± 9.7 kg, greater than 5% in 66.9%. This was higher than that achieved in the SUSTAIN (4) trials, although the initial weight in our study was slightly higher: 98.48 ± 16.6 vs. 89–95 kg in SUSTAIN 1–5 and 7. Weight loss was more notable if T2DM had been evolving for less than 5 years, which demonstrates the usefulness of using semaglutide early due to its repercussion on good control of T2DM, the absence of vascular complications, the positive effect on adherence to treatment, and the improvement of quality of life (21). When analyzing the combined variable of HbA1c <7% and weight loss >5%, this result was obtained in 63.9% of the GLP-1RA-naïve participants and 51.1% of the GLP-1RA-experienced group. Although the difference did not reach significance, the good results in just 2 years again highlight the importance of using semaglutide in all participants who do not achieve individualized metabolic control goals, assessed every 3–6 months from diagnosis. We must reflect on whether, in this case semaglutide, is the most effective treatment for the global approach to T2DM (18).

Other risk factors that showed improvement were SBP and DBP and the lipid profile of both LDL cholesterol and triglycerides, with a degree of control similar to that achieved in SUSTAIN 1–5 and 7. However, notably, throughout the follow-up, the prescribing physicians were free to modify the doses and number of medications for both hypertension and cholesterol. We have not analyzed the weight of these modifications in terms of achieving better objectives.

The proportion of participants who abandoned semaglutide at the beginning of the study (6.6%) was lower than that reported in SUSTAIN 1–7, which was over 11% (22). Dropouts were mainly due to gastrointestinal side effects, in the form of persistent nausea and diarrhea. In our study, 24 participants were discharged before the end of the follow-up period for achieving their individual metabolic control goals, which supports the potential early improvement of semaglutide in T2DM.

One of the big differences from SUSTAIN is that these clinical trials did not include data on participants who switched to OWS from another GLP-1RA. In the SURE program, this change produced an additional HbA1c-lowering effect of -0.7% and -3.7 kg of weight. The study by Hansen et al. found a reduction in HbA1c of -0.6% and in weight of -3.2 kg. In our study, the reduction was greater, with a decrease in HbA1c of -0.74% and in weight of -7.65 kg. As in other publications, the reasons for switching were failure to meet individual HbA1c or weight targets, improve cardiovascular status, improve adherence (once weekly vs. daily), or delay treatment intensification (23). Other reasons that could influence the improvement when switching from GLP1-AR could be different pharmacodynamic effects given the substantially varying biochemical properties and thereby a different effect on appetite, satiety, gastric emptying and insulin compared to the respective GLP-1 analogues (24).

The SUSTAIN trials also did not examine some variables analyzed in our study, such as the increase in hypoglycemic medication during the study, which was significantly higher in GLP-1RA-naïve participants, fundamentally at the expense of the association of SGLT2-i. They also did not examine the decrease in the number of basal and rapid insulin units, which occurred in our study in both the GLP-1RA-naïve group and the GLP-1RA-experienced group.

Our study is not immune to limitations, attributable to its observational nature. The data were collected as part of routine clinical practice rather than through mandatory assessments at a predetermined time, which could affect data integrity. In addition, the study did not include a comparison arm, meaning that the proportion of changes observed due to semaglutide, spontaneous variation, or study effect could not be assessed. There were also no validated quality of life surveys.

In conclusion, and beyond the figures, in the cohort of participants that we have studied, it was found that the treatment of DM2 with weekly semaglutide in a real-life study, and 24-months follow-up, significantly improved glycemic control and weight loss, corroborating the results found in randomized clinical trials. In the subgroup study, GLP-1RA-naïve participants had significantly better results in glycemic control and weight loss than GLP-1RA-experienced participants. These real-life results support the early use of semaglutide in T2DM as the drug of choice for metabolic control and other risk factors for developing atherosclerosis.

## Data availability statement

The original contributions presented in the study are included in the article/supplementary material. Further inquiries can be directed to the corresponding author.

## Ethics statement

The studies involving human participants were reviewed and approved by the Institutional Research Ethics Committee of Hospital Costa del Sol. The patients/participants provided their written informed consent to participate in this study.

## Author contributions

MDGdeL, JPG-M, BA-B, AIP-M and LMP-B contributed to the conception, design of the work the acquisition, interpretation of data, writing-original draft preparation, writing-review and editing, and supervision. MDGdeL, JPG-M, BA-B, AIP-M, FR-R and LMP-B contributed to the acquisition of data and revised the work. FR-R contributed to interpretation of data, writing-review and editing, and supervision. All authors read and approved the final manuscript. All authors meet the criteria for authorship stated in the Uniform Requirements for Manuscripts Submitted to Biomedical Journals. 

## Conflict of interest

The authors declare that the research was conducted in the absence of any commercial or financial relationships that could be construed as a potential conflict of interest.

## Publisher’s note

All claims expressed in this article are solely those of the authors and do not necessarily represent those of their affiliated organizations, or those of the publisher, the editors and the reviewers. Any product that may be evaluated in this article, or claim that may be made by its manufacturer, is not guaranteed or endorsed by the publisher.
